# Data supporting UCrad and CGM, two novel methodologies for radiological impacts in Life Cycle Assessment

**DOI:** 10.1016/j.dib.2019.104857

**Published:** 2019-11-26

**Authors:** Andrea Paulillo, Roland Clift, Jonathan Dodds, Andrew Milliken, Stephen Palethorpe, Paola Lettieri

**Affiliations:** a Department of Chemical Engineering, University College London, Torrington Place, London, WC1 E7JE, United Kingdom; b Centre for Environment and Sustainability, University of Surrey, Guildford, Surrey, GU2 7XH, United Kingdom; c National Nuclear Laboratory, Workington, Cumbria, CA14 3YQ, United Kingdom; d Ardskell, Embleton, Cockermouth, Cumbria, CA13 9YP, United Kingdom

**Keywords:** Life cycle assessment, Characterisation factors, Radionuclides, Radiological impacts, Ionising radiations, Nuclear waste, Risk assessment

## Abstract

Radiological impacts are often disregarded in Life Cycle Assessment (LCA) due to the lack of a standard and comprehensive framework for including the impacts of radionuclides alongside other emissions from industrial processes. This data article is related to the research articles “Radiological Impacts in Life Cycle Assessment. Part I: General framework and two practical methodologies” [1] and “Radiological Impacts in Life Cycle Assessment. Part II: Comparison of Methodologies” [2], which introduced two practical methodologies for assessing the radiological impacts in LCA; these are UCrad and the Critical Group Methodology (CGM). This article reports the characterisation factors, for routine direct discharges and releases from nuclear waste disposed in a geological disposal facility, obtained from both methodologies. The article also reports the underlying data supporting the methodologies and the analysis carried out in the related research articles.

**Table:** Specifications Table

Subject	Environmental Engineering
Specific subject area	Radiological impacts in Life Cycle Assessment
Type of data	Excel spreadsheets.
How data were acquired	Database for radionuclides properties is based on literature data; characterisation factors are obtained from UCrad and the Critical Group Methodology (CGM), and log-deviations are obtained from the analysis described in the associated research article.
Data format	Raw data
Parameters for data collection	Hierarchical approach for data selection used for radionuclides properties database.
Description of data collection	The hierarchical approach starts from the most comprehensive source, continuing to less comprehensive sources only for data not available in a preferred source.
Data source location	Department of Chemical Engineering, University College London, United Kingdom
Data accessibility	With the article
Related research article	A. Paulillo, R. Clift, J. Dodds, A. Milliken, S. Palethorpe, P. Lettieri, Radiological Impact Assessment in Life Cycle Assessment. Part I: General framework and two practical methodologies, Sci. Total Environ. (2019).

**Table:** 

**Value of the Data** • The database presents a comprehensive collection of physico-chemical properties of radionuclides; characterisation factors enable assessment of radiological impacts in LCA; log-deviations represent radionuclide-specific impact factors deviations between UCrad and CGM. • Data is primarily of interest to LCA practitioners and LCIA (Life Cycle Impact Assessment) methodology developers. • Data can also be used for comparison with other methodologies, for improving radiological impacts assessment in LCA, and for further analysis of the proposed methodologies.

## 1 Data

The data reported in this article includes i) a database of the radionuclides physico-chemical properties and dose/risk conversion factors; i) the characterisation factors obtained from the methodologies, and iii) their log deviations. The data is reported in three separate excel workbooks.

### 1.1 Database of radionuclides properties

The database of physico-chemical properties and dose/risk conversion factors of radionuclides uses an excel spreadsheet adapted from the USEtox model [3]. For each radionuclide, the database includes data such as half-life, partitioning and bio-accumulation coefficients and effective dose factors that is used in the Fate, Exposure and Effect modules of UCrad and CGM [1]. As described in Paulillo et al. [1], the database was compiled using a hierarchical approach for data selection because several authoritative sources were available in the literature. The excel spreadsheet reports selected as well as unselected datasets.

### 1.2 Characterisation factors

Characterisation factors obtained from UCrad and CGM are reported for direct discharges to air, freshwater and seawater, and for emissions arising from nuclear waste disposed in a geological repository. They are expressed in absolute terms as yearly risk per Becquerels (Bq) released, and in relative terms as Bq equivalent per Bq released, obtained by dividing the characterisation factor for each nuclide by that of a reference substance emitted to a specific environmental compartment. The references substances are uranium-235 (U235) emitted to air and uranium-238 (U238) from I-LLW (Intermediate-Low Level Waste). CGM factors for direct discharges are reported for four distances of the critical group, namely 1, 100, 1000 and 10 000 km, whilst only one set of CGM factors is provided for emissions from nuclear waste.

### 1.3 Log deviations

The article also includes log deviations between UCrad and CGM characterisation factors for operational emissions to three receiving environmental media, i.e. air, freshwater and seawater, and for four distances from source to receptor modelled using the CGM approach. Log deviations are reported separately for high and low values of half-life, with the threshold between high and low values being equal to 7e+07 s (see Ref. [2]).

## 2 Experimental design, materials, and methods

Paulillo and colleagues proposed a framework for integrating radiological impacts into LCA [1] that consists of a Fate, Exposure and Effect Module. UCrad and CGM share the same Exposure and Effect Modules but employ different Fate Modules to estimate the concentration of radionuclides, following their release, in different environmental media. UCrad applies the multimedia compartment approach proposed by Mackay [4], whilst CGM use Human and Environmental Risk Assessment approaches, adapted from Ref. [5] for routine direct discharges and from Ref. [6] for releases from nuclear wastes disposed in a geological disposal facility. From the environmental concentrations estimated by the Fate Module, the Exposure Module predicts the amount of ionising radiation absorbed by human beings and the Effect module converts the predicted exposures into an effective dose, measured in Sieverts (Sv). The dose may also be converted into a risk metric for detrimental effects.

Log deviations are defined as the logarithms to base 10 of the ratios of the characterisation factors for each radionuclide. They were used in Refs. [1,2] to quantify the average difference between sets of characterisation factors of CGM and the reference set of Ucrad.

## References

[1] Paulillo A., Clift R., Dodds J.M., Milliken A., Palethorpe S.J., Lettieri P. (2019). Radiological impact assessment in life cycle assessment. Part I: general framework and two practical methodologies. Sci. Total Environ..

[2] Paulillo A., Clift R., Dodds J.M., Milliken A., Palethorpe S.J., Lettieri P. (2019). Radiological impact assessment in life cycle assessment. Part II: Comparison of methodologies. Sci. Total Environ..

[3] Rosenbaum R.K., Bachmann T.M., Gold L.S., Huijbregts M.A.J., Jolliet O., Juraske R., Koehler A., Larsen H.F., MacLeod M., Margni M., McKone T.E., Payet J., Schuhmacher M., Van De Meent D., Hauschild M.Z. (2008). USEtox - the UNEP-SETAC toxicity model: recommended characterisation factors for human toxicity and freshwater ecotoxicity in life cycle impact assessment. Int. J. Life Cycle Assess..

[4] Mackay D. (2001). Multimedia Environmental Models: the Fugacity Approach.

[5] IAEA (2001). Generic Models for Use in Assessing the Impact of Discharges of Radioactive Substances to the Environment.

[6] NDA (2010). Geological Disposal: Generic Post-closure Safety Assessment. http://scholar.google.com/scholar?hl=en&btnG=Search&q=intitle:Geological+Disposal+Generic+Post-closure+Safety+Assessment#1.

