# Corrigendum to “Data supporting UCrad and CGM, two novel methodologies for radiological impacts in life cycle assessment” [Data in Brief 28 (2020) 104857]

**DOI:** 10.1016/j.dib.2025.111719

**Published:** 2025-05-23

**Authors:** Andrea Paulillo, Roland Clift, Jonathan Dodds, Andrew Milliken, Stephen Palethorpe, Paola Lettieri

**Affiliations:** a Department of Chemical Engineering, University College London, Torrington Place, London WC1 E7JE, United Kingdom; b Centre for Environment and Sustainability, University of Surrey, Guildford, Surrey GU2 7XH, United Kingdom; c National Nuclear Laboratory, Cumbria, Workington CA14 3YQ, United Kingdom; d Ardskell, Embleton, Cockermouth, Cumbria, CA13 9YP, United Kingdom

The authors regret that there was an error in the characterisation factors of the CGM methodology. Specifically, this error refers to the factors in the sheet “CGM – direct discharges” for a distance of 10,000 km and expressed in relative terms as Bq U235 air-equiv. per Bq released. The factors were erroneously normalised using Br82 rather than U235. The corrected factors are included in this publication.

The authors would like to apologise for any inconvenience caused.

